# Signaling Allyship Via EARS: An Investigation of a Novel Allyship Framework Among Black Women in Maternal Health Settings

**DOI:** 10.1007/s40615-025-02603-6

**Published:** 2025-08-14

**Authors:** India R. Johnson, Veronica S. Derricks, Sacha Sharp, Francesca Williamson, Evava S. Pietri, Jesse Stewart, Jasmine D. Johnson, Melissa D. Klitzman

**Affiliations:** 1 Department of Psychology, Indiana University, 402 N. Blackford Street, 100 A, Indianapolis, IN 46202-3275 USA; 2 Department of Psychology and Neuroscience, University of Colorado – Boulder, Boulder, CO USA; 3 School of Medicine, Indiana University, Indianapolis, IN USA; 4 University of Michigan Medical School, Learning Health Sciences, Ann Arbor, MI USA

**Keywords:** Patient-provider communication, Black maternal health, Racial allyship

## Abstract

Low-quality patient-provider interactions contribute to and further exacerbate Black women’s poor maternal health outcomes. Perceiving one’s provider as an *ally*, or someone that acknowledges and addresses racial inequities can potentially transform the quality of patient-provider encounters; yet little research has investigated how to effectively convey racial allyship among Black women. In the present investigation we examined the utility of a novel racial allyship framework, **EARS** (i.e., **E**mpowerment via collaborative decision-making, **A**pproaching racism dialogue, **R**ecognition of systemic racial inequities, and **S**ocial support for racism-related stress), a collection of interrelated patient care objectives to convey provider allyship among Black women in maternal health settings. We conducted a cross-sectional, retrospective survey with Black women that had received prenatal care in the United States since 2020 (*N* = 285). To gauge patient-provider interaction quality, participants reported perceptions of prenatal provider identity-safety (i.e., beliefs their identities were valued by their prenatal provider). Participants also reported pregnancy complications, and critically, perceptions their prenatal provider was an ally – as defined by our EARS framework. We found that perceptions one’s prenatal provider was an ally significantly predicted greater prenatal provider identity-safety and fewer self-reported pregnancy complications, even when controlling for established predictors of provider evaluations (e.g., patient-provider race concordance, provider communication quality) and pregnancy-related complications (e.g., maternal age, number of prenatal visits). The present investigation documents the utility of the EARS framework, and, for the first time, links provider allyship to an improved pregnancy-related health outcome among Black women.

## Signaling Allyship Via EARS: An Investigation of a Novel Allyship Framework Among Black Women in Maternal Health Settings

“In the U.S., Black women are nearly three times more likely to die during or after childbirth than their White counterparts…Being heard and appropriately treated was the difference between life or death for me; I know those statistics would be different if the medical establishment listened to every Black woman’s experience.” – Serena Williams [1].

The above quote was included in Williams’ 2022 essay detailing her harrowing birthing experience following the delivery of her oldest child, Olympia Ohanian. As Williams alludes, the maternal mortality rate among Black women in the U.S. is among the highest of all racial groups, with a rate of 50.3 deaths for every 100,000 live births [2]. Notably, in her essay, Williams, described repeatedly requesting medical intervention to address her history of blood clots, a persistent request that ultimately saved her life. Relative to White women, Black women in the United States are two to three times more likely to die from pregnancy-related complications. Of note, this heightened risk of maternal mortality among Black women spans all income and education levels [3]; and, critically, most pregnancy-related deaths are preventable [4]. Although multiple factors contribute to such disparities, like Williams, Black women routinely describe their concerns being ignored during medical encounters [5, 6]. Williams’ experience captures the importance of high-quality patient-provider interactions where Black women feel heard.

High-quality clinical encounters are associated with perceiving one’s provider positively and crucially, better patient health outcomes [7–9]. A burgeoning body of literature theorizes [10–12] that perceiving one’s provider as an *ally*, or an individual that acknowledges and acts to address racial inequities [12–14] can potentially transform the quality of patient-provider encounters and pregnancy-related outcomes among Black women patients. Moreover, exploring provider allyship supports recent calls to address anti-Black bias and center racial equity in patient-centered care [15]; yet, empirical research investigating approaches to promote allyship among Black women in maternal health encounters is scant.

To address this knowledge deficit, in the present work we investigated the utility of a novel approach for maternal care providers to signal their racial allyship among Black women in patient-provider encounters, which we refer to as the EARS framework. **EARS** is an acronym for a set of interrelated clinical care objectives – **E**mpowerment via collaborative decision-making, **A**pproaching racism dialogue, **R**ecognition of systemic racial inequities, and **S**ocial support for racism-related stress – aimed at signaling provider allyship and in turn, transforming the quality of patient-provider interactions. Notably, the EARS framework builds upon existing models of healthcare delivery grounded in cultural competence [16, 17] by centering two essential aspects of racial allyship: acknowledging and addressing racial inequities [14, 18].

To demonstrate the utility of our EARS framework, in the present investigation we examined whether perceiving one’s pregnancy care provider as an ally – as defined by our EARS framework – predicted Black women’s evaluation of their patient-provider encounters with their prenatal provider, and self-reported pregnancy complications, a key predictor of severe maternal morbidity and mortality [19].

## Identity-Safety & Allyship in Patient-Provider Interactions

Black women face unique challenges whilst navigating maternal healthcare systems. Structural racism fosters and reinforces racial discrimination within maternal and pregnancy care via systemic factors such as differential quality and access to maternal care, that in turn, perpetuates race-based discriminatory beliefs and practices [20, 21]. As such, Black women report the highest rates of mistreatment during maternal and pregnancy care in the United States relative to other racial/ethnic women [22]. Repeated interpersonal experiences with discrimination and mistreatment embodies an artifact of structural racism that ultimately triggers reductions in *identity-safety*, or beliefs that individuals possessing one or more of your identities are treated with respect and fairness in a particular context [23, 24].

Identity-safety is a multi-faceted collective set of concerns [24] and comprises several identity-relevant constructs (e.g., beliefs that members of your identity groups will be treated fairly, trust in others, feelings of belonging) [25]. Moreover, although identity-safety has been indexed in health settings by assessing several outcomes related to patient-provider interaction quality (e.g., provider trust) [11, 12], the construct also captures beliefs regarding the likelihood for identity-based mistreatment and devaluation [26]. Critically, feeling a sense of identity-safety in clinical encounters is associated with continuous engagement in healthcare, adherence to provider recommendations, and positive perceptions of patient-provider encounters [12]; all robust predictors of patient health outcomes [8, 9].

Central to the present investigation, integrating provider allyship in clinical encounters is an underexplored, yet promising strategy to transform perceptions of clinical encounters and pregnancy outcomes. Nascent literature finds that allyship perceptions are positively associated with identity-safety among Black women [27–29]. Although the potential for provider allyship to improve patient-provider interactions has been theoretically proposed [10, 11, 30, 31] the benefits of allyship among Black women in maternal care has yet to be empirically tested. Moreover, in contrast to interventions that place the onus on the patient to motivate greater engagement in care [32, 33] focusing on provider allyship shifts the responsibility of behavior change to medical providers to facilitate improved encounters. Conveying that one is an ally is highly interpersonal in nature [13] and requires consistent relationship building to signal one’s sincere commitment to dismantling inequities [14, 18, 34]. In the United States, birthing persons have approximately 15 prenatal visits for uncomplicated pregnancies, with even more visits for those with complications [35, 36]. Although equitable access to prenatal care remains an ongoing and pressing need [21], prenatal care encounters present an opportune time for providers to convey allyship. Despite, existing work has yet to introduce an effective approach to embed racially equitable practices into prenatal encounters to improve the quality of patient-provider interactions and patient outcomes.

## The EARS Framework

Fortunately, the EARS framework addresses this gap in scholarship and offers a novel approach to racial allyship in clinical encounters. Intergroup research examining racial allyship from the vantage point of Black individuals has conceptualized allyship as having two key ingredients: acknowledging racial inequities and acting to address these inequities (i.e., acknowledgement and action, respectively) [14, 18]. Provider racial allyship in clinical encounters is an emergent area of scholarship; thus, we conducted a literature review to home in on how acknowledgement and action were best represented in the context of Black women’s maternal care. As such, we drew heavily from research detailing Black women’s pregnancy care encounters with their providers in the United States, exclusively focusing on qualitative work including Black women in their study samples[31, 37–39].^1^ In this regard, the development of our EARS framework was not only guided by theorizing on racial allyship in intergroup settings, but notably, relied heavily on research centering the voices of Black women.

The first aim of our EARS framework – *Empowerment via collaborative decision-making* – was informed by research detailing Black women’s experiences of being ignored and unheard during clinical encounters. Indeed, a wealth of literature highlights that prenatal providers often fail to listen and take Black women’s concerns seriously [5, 39–41]. Relatedly, several studies point to Black women feeling as though their voice and perspectives are excluded from their health decisions, and that prenatal encounters lack shared decision-making [31, 38]. As such, we conceptualized empowerment via collaborative-decision making as providers explicitly engaging their patient in their care by 1) listening to their patients, and 2) expressing to their patients that they are equal partners when it comes to their healthcare decisions.

*Approaching racism dialogue* and *Recognition of systemic inequities* – the “A” and “R”, respectively, in our EARS framework are both informed by an abundance of scholarship denoting that Black individuals describe racism and identity-based mistreatment as common occurrences in their day-to-day lives [42–45]; yet pregnancy providers seldom initiate conversations about race or racism [46, 47]. Likewise, many providers adopt a colorblind approach to patient care [48, 49] and fail to acknowledge the role systemic racism plays in maternal health outcomes [37, 50–52]. Recognition that racial health disparities are a byproduct of systemic inequities is characterized as a necessary precursor of racial allyship among Black individuals [14, 18, 30]. Qualitative work detailing Black women’s perspectives denotes their preference for person-centered care that acknowledges their lived experiences (e.g., racism) [40, 53, 54], and provider communicative practices that go beyond “standard” models-of-care, and instead implement racially-safe practices [37, 41]. Critically, integrating racism dialogue into clinical encounters has the potential to facilitate relationship building and trust between providers and Black patients [4, 5, 47]. Thus, we conceptualized approaching racism dialogue as normalizing conversations about racism in clinical encounters, and recognition of systemic inequities as demonstrating an awareness of how systemic racism contributes to maternal health disparities and Black women’s lived experiences.

*Social support for race-related stress* is the final aim of the EARS framework and stems from investigations noting that Black women often feel it is their responsibility alone to navigate the inherent stress and trauma associated with racism [55]. Social support can serve as a buffer against the negative health-related effects of stress [56], and importantly, racism-specific social support that conveys a mutual understanding of racial discrimination can be beneficial for Black individuals [47, 57–59]. Moreover, collaboratively identifying strategies to address race-related stress with patients can signal to Black women patients a provider is invested in individualized person-centered care [40, 53]. Hence, we conceptualized social support for race-related stress as validating patients’ experiences and working alongside patients to find effective strategies to mitigate such racism-related stress.

Critically, we posit that the EARS framework is an interrelated set of clinical aims that are a unique manifestation of patient-centered care that enhances the quality of Black women’s medical encounters with their provider above and beyond established provider and patient characteristics associated with better-quality patient-provider interactions and patient outcomes. For example, relative to racially concordant encounters, racially discordant encounters (i.e., interactions between patients and providers who do not share a racial identity) tend to be characterized as having lower-quality communication [60, 61]; and, access to Black physicians is associated with better health outcomes among Black patients [62]. Similarly, though the benefits of gender concordant care (i.e., a shared gender identity between patients and providers) has found inconsistent evidence that gender concordant encounters produce higher quality patient-provider interactions, some investigations suggest improved outcomes for women patients receiving care from women physicians [63, 64]. Thus, in the present study, we controlled for the racial identity and gender identity of participants prenatal provider, as well as participants’ perceptions of their provider’s communication quality to demonstrate the unique explanatory power of our EARS framework.

In a similar vein, to isolate provider allyship as a unique predictor of participants’ self-reported pregnancy complications, we also controlled for participants gestational age at delivery, maternal age, and number of prenatal visits – all established predictors of pregnancy complications [65]. Moreover, daily experiences of interpersonal racial discrimination are a chronic psychosocial stressor among Black women [44], and such experiences throughout pregnancy are associated with a range of adverse maternal outcomes [66]. To ensure provider allyship significantly predicted our outcomes of interest despite Black women’s everyday experiences with racism, we also measured and controlled for participants’ experiences with interpersonal racism throughout their pregnancy. We hypothesized that perceiving one’s provider as an ally – as defined by the EARS framework – would be associated with more positive evaluations of patient-provider interaction quality, and fewer self-reported pregnancy complications, independent of provider and patient characteristics associated with improved patient-provider interaction quality and reduced rates of pregnancy-related complications.

## Method

### Participant Recruitment and Data Collection

Participants were recruited via the online data acquisition platform, CloudResearch [67], which utilizes convenience sampling to assist researchers with targeted recruitment of specific populations. Participants were paid $2.00 for a single-session 10-min study (approximately $12.00/hour). We exclusively recruited United States-born Black women that met the following criteria: 1) were not pregnant at the time of study completion, 2) indicated they have had at least one pregnancy resulting in the delivery of a single baby since 2020, and 3) attended at least one prenatal appointment within the United States for their pregnancy.

### Procedure & Outcome Measures

Participants were instructed that the study was investigating the experiences of Black women who received prenatal care within the United States. The instructions noted we were specifically interested in the quality of their interactions with their healthcare provider and the definition of a healthcare provider was provided for participants (i.e., “A healthcare provider is any person who provided healthcare during your prenatal visit(s) and could be your physician (for example your obstetrician), a different physician in your healthcare center, a physician assistant, a midwife, or a nurse”. Participants were instructed to answer questions based on the provider they most often saw during prenatal visits and were also prompted to do so before each measure relevant to prenatal provider perceptions.

The end of the survey included a demographic questionnaire with conventional sociodemographic characteristics including current age (years), annual income (dollars), marital status (single, married, separated, divorced, widowed, living with a partner, I prefer not to say), highest education level (less than high school, high school diploma or equivalent, associate’s degree, bachelor’s degree, advanced degree), employment status (employed at least part-time), and insurance status (yes, no, prefer not to disclose). Participant demographic characteristics can be found in Table 1.

**Table 1:** Sociodemographic characteristics of participants (*n* = 285)

Characteristic	*n (%)*
Age, mean = 29.78 years (*n* = 251)	
Marital Status	
Single	128 (44.9)
Married	77 (27.0)
Separated	4 (1.4)
Divorced	5 (1.8)
Living with partner	65 (22.8)
Widowed	6 (2.1)
Highest education level	
Less than high school	15 (5.3)
High school diploma or equivalent	165 (57.9)
Associates	55 (19.3)
Bachelors	37 (13.0)
Advanced degree	13 (4.7)
Annual income	
< $20,000—$39,999	151(53.0)
$40,000—$59,999	53 (18.6)
$60,000—$79,999	35 (12.3)
$80,000 -$99,999	18 (6.3)
$100,000	19 (6.7)
Prefer not to disclose	9 (3.2)
Employed at least part-time	184 (64.6)
Have health insurance	259 (90.9)

*Note*. Sample size is equal to 285 for all sociodemographic characteristics, with the exception of age

### Provider Identity-Safety

We operationalized patient-provider interaction quality by indexing provider identity-safety. Our measure of identity-safety has demonstrated excellent reliability and construct validity in previous work [11, 26] and importantly, has been utilized to index patient-provider interaction quality among Black-identified participants [12]. Participants completed an 11-item measure of identity-safety comprised of provider trust, adherence to the provider’s treatment plan, and interest in seeing the provider again in the future – all predictors of patient health outcomes [9, 68]. Each item referenced participant’s prenatal provider (e.g., “I trusted my prenatal provider to treat me fairly”). Participants responded to all items utilizing a seven-point Likert scale (1 = strongly disagree, 2 = disagree, 3 = somewhat disagree, 4 = neither agree nor disagree, 5 = somewhat agree, 6 = agree, 7 = strongly agree), and we averaged participants’ responses to the items, with higher scores indicating greater identity-safety (α = 0.95).

### Pregnancy Complications

Participants’ were provided with seven of the most common pregnancy complications identified by the National Institute of Child Health and Human Development [19] and indicated whether they experienced any during their pregnancy and/or delivery. The provided options were high blood pressure, gestational diabetes, pre-eclampsia, preterm labor (i.e., labor that begins prior to 37 weeks of pregnancy), stillbirth, pregnancy-related depression and/or anxiety, and infections (including, but not limited to sexually transmitted infections). Participants were also given the option to self-report any other complications beyond the list provided. Participants’ responses were summed with higher numbers indicating greater complications.

### Primary Predictor Variable

#### Provider Allyship

Our 13-item measure of provider allyship mapped onto our EARS framework (see Table 2 for all items; e.g., “My provider tailored my pregnancy care to my needs as a Black woman”), and collectively, captured the extent to which participants’ prenatal provider engaged in behaviors identified by Black women as reflecting a commitment to sincere racial allyship in maternal care settings. Items included those from previously published work [12, 69], and research centering the voices of Black women during their pregnancy and delivery care [5, 6, 31, 39]. Participants responded to all items using the same scale as that for identity-safety (e.g., a seven-point Likert scale), and we averaged participants’ responses to the items, with higher scores indicating greater provider allyship (α = 0.93).

**Table 2:** Prenatal provider allyship items via the EARS framework

Signaling allyship via EARS items	Percent of participants that reported somewhat agree or higher (*n* = 285)
1. My provider and I worked together to identify solutions to any concerns I raised	78.0
2. My provider actively listened to any concerns I shared	77.5
3. My provider valued my perspective when making decisions about my pregnancy and health	77.5
4. My provider tailored my pregnancy care to my needs as a Black woman	54.7
5. My provider cared about issues related to Black women’s reproductive health	54.4
6. My provider made a genuine effort to support Black women’s maternal health	53.9
7. My provider was aware of racial health disparities in Black women’s maternal health outcomes	50.0
8. My provider adopted practices that improve reproductive maternal health outcomes for Black women	47.5
9. My provider considered how my experiences as a Black woman shaped my pregnancy experiences	45.4
10. My provider was comfortable answering questions that related to race and racism in maternal healthcare	44.2
11. My provider regularly checked in about how I was coping with any race-related stress during my pregnancy	37.8
12. My provider encouraged me to talk about any stress I was feeling because of racial discrimination in my day-to-day life	37.3
13. My provider talked about the role of systemic racism in producing poor health outcomes for Black mothers	29.8

### Control Variables

To ensure perceptions of prenatal provider allyship predicted identity-safety and pregnancy complications above and beyond other established predictors of greater quality patient-provider interactions and patient outcomes, we measured several control variables.

#### Provider Race

Participants reported the race of the prenatal provider they most often saw, and we dummy coded whether the provider was racially concordant (i.e., Black) or discordant (i.e., racially concordant = 1, racially discordant = 0).

#### Provider Gender

Participants also reported the gender of the prenatal provider they most often saw; like provider race, we dummy coded whether the provider was gender concordant (i.e., female) or discordant (i.e., gender concordant = 1, gender discordant = 0).

#### Provider Communication Quality Scale

Patient-provider communication quality was a subset of items taken from the Communication Quality Scale (CQS) utilized in previous research to examine the experiences of racial and ethnic minority women in maternity care [70]. Of note, because the present investigation focused on prenatal care experiences, we only utilized the seven items that referred to experiences during prenatal visits (e.g., “My prenatal care provider seemed rushed”). Participants responded to all items using the same scale as that for identity-safety (e.g., a seven-point Likert scale), and we averaged participants’ responses to the items, with higher scores indicating greater provider communication quality (α = 0.86).

#### Everyday Discrimination Scale

Participants also completed the Everyday Discrimination Scale (EDS) [71], and we adapted the scale’s format to assess frequency of discrimination during their pregnancy. Specifically, the nine items were presented with the prompt “In your general day-to-day life during your pregnancy, how often did any of the following things happen to you?” and a sample item included “You were treated with less respect than other people were”. Response options included 1 = never, 2 = once during my pregnancy, 3 = a few times during my pregnancy, 4 = at least once a month during my pregnancy, 5 = a few times a month during my pregnancy, and 6 = almost every day. Responses were averaged with higher scores reflecting greater frequency of discrimination during pregnancy (α = 0.94).

#### Gestational Age at Delivery

Participants self-reported the gestational age of their baby at time of delivery. Specifically, participants were instructed to report the number of weeks and days, separately. We converted the number of weeks reported to days and added this number to the remaining days reported by participants. Hence, gestational age was a continuous variable reported in days where higher numbers indicated greater gestational age at time of delivery.

#### Maternal Age

Participants reported their age at time of delivery in years. Maternal age was treated as a continuous variable where higher numbers reflected more advanced maternal age.

#### Number of Prenatal Visits

Participants estimated the number of prenatal visits they attended throughout their pregnancy. The number of visits was also treated like a continuous variable and higher numbers reflected a greater number of self-reported visits.

## Results

### Participant Characteristics

Our sample comprised of 285 participants between the age of 18 and 52 years old (age_*mean*_ = 29.78 years, age_*SD*_ = 7.45 years). All 285 participants (100.0%) identified as Black women and were born in the United States. As shown in Table 1, most participants were single (128; 44.9%), had at least a high school education or equivalent (165; 57.9%), had an annual income of $39,999 dollars or less (151; 53.0%), were employed at least part-time (184; 64.6%), and had health insurance (259; 90.9%).

### Analytic Approach & Data Analysis

Given the novelty of our provider allyship items, we first conducted an exploratory factor analysis (EFA) to examine whether our 13-item scale indexing provider allyship comprised a single construct. Because our goal was to examine whether a one-factor solution was acceptable, we utilized a principle components analysis for extraction [72] with Kaiser Normalization to enhance interpretability of the factor structure. The Kaiser–Meyer–Olkin measure revealed a sampling adequacy of 0.942, indicating excellent suitability of the data for factor analysis. Moreover, the Bartlett’s Test of Sphericity was significant χ^2^(78) = 2481.92, *p* < 0.001, supporting the factorability of the correlation matrix.

Our EFA revealed a two factor solution with an eigenvalue of 7.44 for the first factor, and an eigenvalue of 1.55 for the second factor. The first factor included items 4–13 (α = 0.87; see Table 2 for items) and accounted for 57.2% of the variance, with factor loadings ranging from 0.69—0.85. The 10 items loading onto the first factor focused on the extent to which the provider utilized race-conscious approaches (e.g., “My provider was comfortable answering questions that related to race and racism in maternal healthcare”). The second factor included items 1–3 (α = 0.94; see Table 2 for items) and accounted for an additional 11.9% of the variance, with factor loadings ranging from 0.55—0.62. The three items centered on the extent to which provider’s listened and worked collaboratively with their patient (e.g., “My provider valued my perspective when making decision about my pregnancy and health”).

Notably, the second factor revealed that many of the items cross-loaded onto the first factor with higher factor loadings than that for the second factor (i.e., ≤ 0.64). Consequently, we conducted a second EFA to examine a single-factor solution. The EFA analyses revealed an eigenvalue of 7.44 that accounted for 57.2% of the variance for all 13 items (α = 0.95) with factor loadings ranging from 0.64—0.85. Because our EFAs revealed a one-factor solution was reliable, and our general pattern of results remained the same when separating our measure into subscales reflecting two-factors, for ease of presentation, we conducted our analyses utilizing the composite score for all 13 items indexing provider allyship.

Turning to our analyses to test our hypotheses, we conducted separate multivariable linear regression models for each outcome measure with the goal of assessing whether perceptions of provider allyship (i.e., our primary predictor) significantly predicted identity-safety and pregnancy complications, respectively. To demonstrate that provider allyship uniquely predicted each outcome, we utilized hierarchical linear regression where prenatal provider allyship was entered as the sole predictor in the first step, and all control variables were added in the second step. We also opted for hierarchical linear regression to examine the amount of variance accounted for in provider identity-safety and pregnancy complications predicted by provider allyship alone (i.e., Step 1) relative when control variables were added as predictors (i.e., Step 2). Descriptive statistics for all variables in the regression model can be found in Table 3 and full regression model statistics are shown in Table 4.

**Table 3:** Descriptive statistics for predictor, outcome, and control variables (*n* = 285)

Variable	*M (SD)*
Prenatal provider allyship	4.46 (1.43)
Prenatal provider identity-safety	5.59 (1.32)
Pregnancy complications	1.51 (0.77)
Communication quality	4.87 (1.44)
Everyday discrimination	2.06 (1.30)
Gestational age (days)	265.11 (18.26)
Maternal age (years)	27.22 (7.74)
Number of prenatal visits	13.49 (9.46)
	*n (%)*
Black prenatal provider	126 (44.2%)
Woman prenatal provider	233 (81.8%)

**Table 4:** Full regression predicting identity-safety in prenatal visits (top), and pregnancy complications (bottom) from prenatal provider allyship as defined by the EARS framework (*n* = 285)

*Identity-safety in prenatal visits*						
Hierarchical Regression Model Fit Measures						
Overall Model Test						
Model	*R*	Adjusted *R*^2^	*R*^2^ Change	*F*	*df*	*p*
Step 1	.62	.39	.39	174.93	(1, 279)	<.001
Step 2	.78	.59	.22	51.76	(8, 272)	<.001
Predictor Regression Coefficients						
	*b*	*SE*	*t*	*p*	*95% CI*	
Step 1						
**Prenatal provider allyship**	**0.575**	**0.043**	**13.23**	**<.001**	**0.489, 0.660**	
Step 2						
**Prenatal provider allyship**	**0.445**	**0.039**	**11.37**	**<.001**	**0.368, 0.522**	
**Prenatal provider communication-quality**	**0.335**	**0.043**	**7.78**	**<.001**	**0.250, 0.419**	
**Everyday discrimination**	**−0.138**	**0.047**	**−2.92**	**.004**	**−0.231, −0.045**	
Gestational age at delivery (days)	0.003	0.003	0.93	.355	−0.003, 0.008	
Maternal age (years)	0.012	0.007	1.80	.074	−0.001, 0.026	
Number of prenatal visits	−0.001	0.003	−0.46	.645	−0.006, 0.004	
Prenatal provider race (1 = racially concordant, 0 = racially discordant)	−0.126	0.108	−1.16	.246	−0.339, 0.087	
Prenatal provider gender (1 = gender concordant, 0 = gender discordant)	−0.175	0.136	−1.29	.199	−0.443, 0.093	
*Pregnancy Complications*						
Hierarchical Regression Model Fit Measures						
Overall Model Test						
Model	*R*	Adjusted *R*^2^	*R*^2^ Change	*F*	*df*	*p*
Step 1	.23	.05	.05	14.85	(1, 279)	<.001
Step 2	.40	.13	.11	6.18	(8, 272)	<.001
	*b*	*SE*	*t*	*p*	*95% CI*	
Step 1						
**Prenatal provider allyship**	**−0.122**	**0.032**	**−3.85**	**<.001**	**−0.184, −0.060**	
Step 2						
**Prenatal provider allyship**	**−0.091**	**0.033**	**−2.76**	**.006**	**−0.156, −0.026**	
Prenatal provider communication-quality	0.030	0.036	−0.47	.641	−0.098, 0.061	
Everyday discrimination	0.058	0.044	0.82	.414	−0.042, 0.101	
**Gestational age at delivery (days)**	**−0.010**	**0.002**	**−3.96**	**<.001**	**−0.014, −0.005**	
Maternal age at delivery (years)	0.004	0.006	0.67	.504	−0.007, 0.015	
**Number of prenatal visits**	**0.004**	**0.002**	**2.03**	**.043**	**0.001, 0.009**	
Prenatal provider race (1 = racially concordant, 0 = racially disconcordant)	−0.091	0.092	−0.99	.323	−0.271, 0.090	
Prenatal provider gender (1 = gender concordant, 0 = gender disconcordant)	−0.126	0.115	−1.10	.271	−0.352, 0.099	

*Note*. Predictors in each model with a *p-value* *<*.05 (i.e., statistically significant) are in bold. Reported coefficients are unstandardized betas with corresponding standard errors and for all predictors we report these values to the thousandth decimal place

### Provider Identity-Safety

Provider allyship significantly predicted provider identity-safety (*p* < 0.001), such that greater allyship was associated with greater identity-safety, even when controlling for variables associated with greater quality patient-provider interactions. Communication quality (*p* < 0.001) was also positively and significantly associated with prenatal identity-safety, while participants’ EDS scores were negatively and significantly related to prenatal identity-safety (*p* = 0.004). All other predictors in the model were non-significant (*p*-values ≥ 0.074).

### Pregnancy Complications

Prenatal provider allyship also significantly predicted participants’ self-reported pregnancy complications (*p* = 0.006), such that greater allyship was associated with fewer reported pregnancy complications, even when controlling for variables associated with greater likelihood of complications. Gestational age (*p* < 0.001) was negatively and significantly associated with complications, such that lower gestational age was associated with greater complications. The number of prenatal visits (*p* = 0.043) and participants’ EDS scores (*p* = 0.025) were positively and significantly related to pregnancy complications, such that more visits and higher EDS scores related to higher numbers of reported pregnancy complications. All other predictors in the model were non-significant (*p*-values ≥ 0.271).

## General Discussion

The present research investigated the efficacy of the EARS framework, a racial allyship framework that entails clinical objectives providers can enact to convey provider allyship, and in turn, transform the quality of patient-provider encounters and pregnancy-related outcomes among Black women. Critically, perceptions that one’s health provider is an ally is associated with greater identity-safety and more positive provider evaluations [12, 14, 69]; yet, research investigating how to effectively signal allyship exclusively among Black women is limited. To address this gap in previous scholarship, we tested whether perceiving one’s prenatal provider as an ally – as defined by our EARS framework – significantly predicted greater identity-safety for the provider and fewer self-reported pregnancy complications.

Ultimately, we found that perceptions one’s prenatal provider was an ally significantly predicted more positive patient-provider prenatal encounters, as well as fewer self-reported pregnancy complications. Moreover, we demonstrated the explanatory power of provider allyship, with provider allyship emerging as a significant predictor even when controlling for established predictors of provider evaluations and pregnancy-related complications. Thus, the present study is among the first to document the utility of provider allyship in the context of Black women’s perceptions of patient-provider interactions, and, for the first time, links provider allyship to an improved pregnancy-related health outcome among Black women. Evaluating one’s physician positively in the context of patient-provider interactions is associated with higher quality clinical encounters [7, 68], and reducing the likelihood of pregnancy complications significantly reduces risk of maternal death [73]. Black women experience worse maternal health outcomes relative to their White counterparts [4], and while a myriad of factors contribute to these disparities, the present work provides initial evidence that the EARS framework is an effective approach to racial allyship that can improve the quality of prenatal encounters and incidence of pregnancy complications among Black women patients.

The present study also highlights the importance of race-conscious clinical strategies in delivery of healthcare services. Indeed, in recent decades, healthcare providers have been encouraged to integrate culturally competent practices into their patient care, as well as consider how their own biases (e.g., implicit bias) can undermine the quality of patient-provider interactions [74]. Furthermore, although culturally competent care is widely recommended in medicine to combat racial health disparities [16, 75], such approaches are often power-neutral, and can inadvertently downplay, or outright ignore, the significance of discussions that center racism and effective cross-racial dialogue in healthcare settings [76]. In contrast, the EARS framework foregrounds acknowledgement of racial inequities and recognition of the harm that systemic racism contributes to Black women’s maternal health outcomes. Moreover, recent intergroup scholarship suggests that interventions that solely focus on one’s personal biases, ironically, do little to motivate action to address personal biases, and rather, interventions considering Black individuals’ discrimination experiences across institutional contexts (e.g., systemic racism) are most likely to encourage action to address systemic *and* personal biases [77]. Hence, the EARS framework goes beyond existing models of culturally competent care, and underscores the importance of racially-conscious care to convey racial allyship in maternal healthcare encounters among Black women.

Finally, the present investigation extends the blossoming body of literature highlighting the benefits of allyship in intergroup settings among Black individuals. Our conceptualization of allyship was informed by scholarship denoting the importance of acknowledgement and action to convey racial allyship [14, 18], and the present study empirically demonstrates the importance of these components to transform the quality of intergroup encounters in maternal health settings. Relatedly, an additional contribution of the present investigation is that our EARS framework provides an effective approach for conveying racial allyship in medical encounters that centers the perspectives of Black women. Indeed, the United States Centers for Disease Control and Prevention *Hear Her Campaign* [22] stresses the importance of clinical care strategies that center the voices of pregnant and postpartum patients to improve the quality of patient-provider interactions. Our translation of racial allyship into the EARS framework was directly informed by the perspectives and voices of Black birthing persons [5, 31, 39]. Hence, akin to scholarship that details approaches and behaviors physicians can adopt to enact anti-racism in clinical care (e.g., [46]), the present work offers empirically validated aspects of allyship that providers can integrate into their clinical care strategies. Thus, the current investigation provides a roadmap for intervenable areas of action (e.g., engaging in discussions about racism and patient-provider collaborative decision-making) to signal allyship and improve the quality of patient-provider interactions and pregnancy-related health outcomes among Black women.

## Limitations and Future Directions

One notable limitation of the present investigation is that our recruitment strategy relied on convenience sampling, which limits the generalizability of the present work. Likewise, to what extent provider allyship is beneficial among Black women outside of the social locations reflected in our sample remains a question for future work. On the one hand, the present investigation answers calls within medicine to center Black women’s voices in efforts to disrupt and dismantle systemic inequities in maternal health [22, 31, 39]. On the other hand, Black women tend to experience poorer maternal health outcomes relative to their White counterparts [4], even when controlling for variables considered a proxy for a broad range of social locations (e.g., education & income) [3, 78]. In short, although our sample of Black women are suitable to test the utility of allyship, future work should recruit Black women across a broader range of social locations and within clinical settings to enhance external validity.

Although the present investigation demonstrates the EARS framework is an effective and novel approach to racial allyship among Black women, we by no means suggest that allyship is a panacea for racist clinical encounters. Racial allyship is an ongoing, and iterative journey that demands awareness of one’s privileged identities [30, 79, 80], as well as regular actions to dismantle racial inequities [34, 50, 81]. Moreover, being an authentic ally is not an identity that is conveniently adopted for clinical encounters [82] and such ingenuine efforts to signal allyship will only backfire and encourage perceptions that a provider is an insincere ally [82, 83]. Providers who wish to act as sincere and committed allies should engage in anti-racist practices in their professional and personal spheres, such as reflecting on their own privileged identities, employing evidence-based practices to address institutional racism in medicine, and consistently practicing cultural humility and culturally responsive care [13, 14, 46, 80, 84]. While engaging in such efforts may mitigate concerns that a provider’s allyship actions are insincere, importantly, provider allyship behaviors alone cannot and will not address racial disparities in maternal health [85, 86]. Future investigations should investigate the efficacy of provider allyship when integrated into multi-level interventions that also target institutional and structural factors [54, 87] (e.g., lack of racially diverse health care workforce, lack of access to maternal care services) contributing to ongoing health disparities.

An additional limitation of the present work is that our investigation of the EARS framework relied on participants’ retrospective recall of their patient-provider interactions, pregnancy complications, and their general pregnancy experiences (e.g., EDS throughout pregnancy). Additionally, obstetric care regularly involves interactions with a variety of prenatal care providers (e.g., nurse practitioners, obstetricians, midwives); yet, in the present work we only examined the perceptions of allyship for a single provider. Although we utilized self-reports in the present studies, notably, our identity-safety index was comprised of constructs that drive actual behavior in clinical encounters (i.e., communication quality and adherence to provider recommendations; [68] [88]). Relatedly, the accuracy of recall for pregnancy complications has found mixed results, with some researchers finding strong agreement [89], and others finding relatively weak agreement [90]. In a similar vein, our decision to provide participants with a list of the most common pregnancy complications may have contributed to underreporting. Future studies should explore how allyship perceptions for multiple prenatal providers (e.g., team-based healthcare delivery) relates to a range of maternal health outcomes as reported in Black women patients’ medical records.

## Conclusion

Although future work is certainly warranted given the identified limitations, the present investigation represents a critical first step in documenting the utility of the EARS framework as an approach to racial allyship that can transform the quality of patient-provider maternal health encounters and patient outcomes among Black women.

## Data Availability

Data for the present investigation is available by request from the corresponding author.

## Footnotes

1 Medline, PsycINFO, CINAHL Complete, and Health Source: Nursing/Academic Edition were searched in July of 2024 using the Boolean search strategy with a single search string: *Black women* AND/OR *pregnancy* AND/OR *health equity* AND/OR *qualitative research*. Additional inclusion criteria included article language (i.e., English), peer-reviewed scholarship, and articles published between January 2004 – July 2024. We excluded duplicate and unrelated articles (e.g., sample outside the United States, did not include majority Black women), and all articles informing our EARS framework are cited in the References.
